# Assessment and Prediction of Adherence to Methotrexate Using Three Self-Report Questionnaires in Patients with Rheumatoid Arthritis

**DOI:** 10.3390/medicina59081446

**Published:** 2023-08-10

**Authors:** Jelena Ceranic, Darija Kisic Tepavcevic, Milan Petronijevic, Marija Milic, Milija Ceranic, Nemanja Rancic, Gorica Ristic

**Affiliations:** 1 Department of Rheumatology and Clinical Immunology, Military Medical Academy, 11040 Belgrade, Serbia; milanpetronijevic@yahoo.com (M.P.); goricaris68@gmail.com (G.R.); 2 Faculty of Medicine, Institute of Epidemiology, University of Belgrade, 11000 Belgrade, Serbia; darijakt@gmail.com; 3 Faculty of Medicine of the Military Medical Academy, University of Defence, 11042 Belgrade, Serbia; 4 Faculty of Medicine, University of Pristina temporarily settled in Kosovska Mitrovica, 38220 Kosovska Mitrovica, Serbia; marijamilic85@gmail.com; 5 Department of Cardiology, Military Medical Academy, 11000 Belgrade, Serbia; drceranic@gmail.com; 6 Centre for Clinical Pharmacology, Military Medical Academy, 11040 Belgrade, Serbia

**Keywords:** prediction, adherence, methotrexate, self-report questionnaires, rheumatoid arthritis

## Abstract

*Introduction:* Methotrexate (MTX) reduces rheumatoid arthritis activity and ameliorates the long-term functional status in these patients. To achieve this aim, patients need to take their medication regularly. Nevertheless, non-adherence to MTX still remains a considerable issue in the management of rheumatoid arthritis. Objective: This study aimed to estimate the adherence to methotrexate in patients with rheumatoid arthritis and to identify specific non-adherence risk factors. *Methods*: A cross-sectional study included 111 patients (mean age 56.2 ± 10.6 years, 78.4% female, and mean disease duration 6 years (3–13)). Three adherence self-assessment questionnaires were used: the Compliance-Questionnaire-Rheumatology (CQR19), the Medication Adherence Reports Scale (MARS-5), and the Visual Analogue Scale (VAS). We also collected demographic data, disease and treatment characteristics, and anxiety/depression estimation results (Hospital Anxiety and Depression Scale, HADS). *Results:* Adherence was identified in 48.6% of patients (COR19), 70.3% of patients (MARS-5), and 82.9% of patients (VAS questionnaire). All three questionnaires displayed a significant positive mutual correlation: CQR19 with MARS-5 and VAS (r = 0.364, r = 0.329, respectively, *p* < 0.001 for both) and between the VAS and MARS-5 scores (r = 0.496, *p* < 0.001). A significant positive prediction was shown for urban residence (0.347 (0.134–0.901), *p* = 0.030) using the MARS-5, female sex (0.264 (0.095–0.730), *p* = 0.010) according to the CQR19, and for a dose of methotrexate (0.881 (0.783–0.992), *p* = 0.036) using the VAS, while negative predictions were shown for comorbidity number (3.062 (1.057–8.874), *p* = 0.039) and depression (1.142 (1.010–1.293), *p* = 0.035) using the MARS-5 and for older age (1.041 (1.003–1.081), *p* = 0.034) according to the CQR19. The use of steroids was a significant positive predictor in all three questionnaires and remained an independent predictor for methotrexate adherence in the multivariate logistic regression. *Conclusions:* We showed non-adherence to methotrexate in a significant number of patients using all three questionnaires. Concomitant steroid therapy emerged as an independent positive predictor for adherence.

## 1. Introduction

Rheumatoid arthritis (RA) is a chronic inflammatory autoimmune disease characterized by symmetric peripheral polyarthritis and progressive joint damage. Disease-modifying antirheumatic drugs (DMARDs) reduce disease activity and radiologic progression and ameliorate the long-term functional status in RA patients [1]. To achieve this aim, patients need to take their medication regularly.

Methotrexate (MTX) is a synthetic DMARD recommended as the first-line treatment in a patient with RA [2,3] due to its safety and efficacy, different methods of application (parenteral and oral), and drug dose titration possibility [4,5]. It can be used as a monotherapy or combined with other synthetic or biological DMARDs [6]. However, not all patients display a good response to MTX, which can be partially attributed to therapy noncompliance [7,8]. Optimal utilization of MTX requires starting therapy immediately and adherence to a prescribed dose and regimen until a satisfactory response is obtained, followed by complying with a maintenance dose. 

Non-adherence is defined as the avoidance of therapy onset, intentional or unintentional disregard for the physician’s recommendations, or non-persistence with treatment [9]. Several studies have been conducted concerning the adherence to MTX in RA patients. The results were inconsistent due to different criteria for establishing adherence, the vast heterogeneity of participant samples, the employment of different types of measurements, and follow-up length. A literature analysis revealed a large variation in adherence, with its rate range spanning from 59% to 107%, where excessive MTX use was also considered an adherence problem [10,11]. In previous studies, the association of certain demographic and disease characteristics, socio-economic status, mental health, and patients’ beliefs regarding medication with adherence to MTX was found [12,13]. Hope et al. concluded that the mild course of the disease, depression absence, patients’ confidence in the efficacy and necessity of the therapy, as well as MTX monotherapy are potential predictors of adherence to MTX [10]. According to the literature, more than 200 variables have an influence on adherence, but none of them showed correlative consistency in different studies [14], indicating that no uniform non-adherence profile could be made [15].

In order to enhance adherence, the priority is to identify individuals displaying non-adherent behavior [16]. Along with the substantial subjective and objective measurement approaches, no strategy is rated as optimal. A multi-method that combines self-assessment and reasonable objective measurements is currently the most adequate procedure to estimate adherent behavior. Still, the most feasible manner of detecting non-adherent patients in clinical practice is a self-reporting method. 

In addition to the need for the better education of patients, there are still numerous issues in adherence improvement [17,18]. Considering the existence of a still-significant number of non-adherent patients, innovative interventions to optimize the use of MTX are needed to improve the disease’s final outcome [19]. Therefore, this research aimed to estimate adherence to MTX and identify specific socio-demographic, clinical, and psychological risk factors for non-adherence. 

## 2. Materials and Methods

### 2.1. Study Patients

This cross-sectional study included patients treated in the Rheumatology Clinic of the Military Medical Academy in Belgrade during 2016 and 2017 who met the following requirements: RA diagnosis based on the classification criteria ACR/EULAR2010 [20], age ≥ 18 years, and prescribed MTX with minimum two months’ continuance. Exclusion criteria were the presence of another connective tissue systemic disease, psychiatric disorders, and antidepressant therapy within the last month, as well as recent infections and surgical procedures. 

This is a single-center study approved by the institutional ethics committee and it conformed to the Declaration of Helsinki. All participants have signed informed consent.

Literature-based questionnaires were used to collect data about patients and disease characteristics. One part of the questionnaire was related to socio-demographic characteristics (sex, age, place of residence, education, employment, and smoking status) and the other to clinical aspects of the disease (disease duration, current MTX dose, maximal MTX dose, therapy side effects, concomitant use of steroids, other DMARDs or biologics, comorbidity presence, frequency of blood laboratory analysis, and the annual number of physician visits). 

The following inflammatory markers were analyzed: erythrocyte sedimentation (ESR) rate according to Westergreen (mm/h), C reactive protein (CRP) using nephelometry (range 0–5 mg/L), and interleukin 6 (IL-6) serum level engaging enzyme-linked immunosorbent assay (ELISA, Bionova, Madrid, Spain) according to the manufacturer instructions (range 0–5.9 pg/mL). 

RA activity was assessed by Disease Activity Score 28-joint count (DAS28-ESR) [21] and Clinical Disease Activity Index (CDAI) [22,23,24]. Disease activity was defined as remission (DAS28 < 2.6, CDAI ≤ 2.8), low disease activity (DAS28 2.6–3.2, CDAI 2.8–10), moderate disease activity (DAS28 3.2–5.1, CDAI 10–22), and high disease activity for DAS28 over 5.1 and CDAI over 22. The functional ability was estimated using a questionnaire for assessing patients’ health status, the Health Assessment Questionnaire (HAQ), more accurately the disability index HAQ-DI [25]. An HAQ score from 0–1 represents mild to moderate difficulties, 1–2 moderate to heavy disabilities, and 2–3 severely heavy disabilities. 

### 2.2. Adherence Assessment

Adherence to MTX therapy was conveyed employing three literature-based questionnaires: the Compliance-Questionnaire-Rheumatology (CQR19), the Medication Adherence Reports Scale (MARS-5), and freely estimated adherence on the Visual Analogue Scale (VAS) ranging from 0 to 10 cm.

The CQR19 [26,27] is composed of 19 statement questions regarding specific barriers in taking prescribed medication and the patient grades the level of agreement according to a Likert scale from 1 to 4 (1 point = strongly disagree, 2 points = disagree, 3 points = agree, and 4 points = strongly agree). Out of the 19 questions, 6 were formulated in negation (numbered questions 4, 8, 9, 11, 12, and 19); therefore, reverse scoring had to be applied. The overall score was obtained by adding up all points and subtracting 19 from the total sum. The new calculated value was divided by 0.57. This ensures that the complete CQR19 result can vary from 0 (no adherence) to 100 (perfect adherence) [26,27,28]. In agreement with published data, a less than 80% score represents low adherence. 

The MARS-5 is an adherence questionnaire developed for patients with different chronic diseases [29]. The questionnaire is composed of 5 questions regarding certain aspects of non-adherent behavior. Each question has multiple-choice answers: 1 = always, 2 = often, 3 = sometimes, 4 = rarely, and 5 = never. The total MARS-5 score can take values from 5 to 25, with a higher rating indicating elevated adherence levels. Different studies suggest that in the majority of measuring scales, a score above 80% is considered satisfactory. Therefore, in this study, patients with MARS-5 scores above 23 were qualified as adherents [30].

The Visual Analogue Scale (VAS) ranges from 0% to 100%. On a scale span from 0 cm to 10 cm, a patient solely determines the level of adherence by marking a number or position that most adequately suits their subjective estimation of compliance to therapy. Values beyond 80% were considered a high level of therapy adherence.

### 2.3. Mental Health Assessment

Mental health assessment was conveyed by the Hospital Anxiety and Depression Scale (HADS) questionnaire, recommended by The National Institute for Health and Care Excellence (NICE) in order to diagnose depression and anxiety [31]. The questionnaire was comprised of 14 questions, with half of them (7) related to anxiety and the other half (7 related) to depression. Each question was answered according to a Likert scale with 4 possible answers (0–3). The overall score for each scale ranged from 0 to 21 and was categorized as 0–7 = normal finding, 8–10 = borderline anxiety/depression, and 11–21 = abnormal, presence of anxiety/depression [32,33]. The specificity of these scores is 0.78 and 0.79 for anxiety and depression, respectively, while score sensitivity values corresponding to anxiety and depression are 0.9 and 0.83 [34]. 

### 2.4. Statistical Data Analysis

Statistical analysis was performed using IBM SPSS version 26.0 (IBM Corp.Released 2019. IBM SPSS Statistics for Windows, version 26.0 Armonk, NY: IBM Corp). Descriptive values were presented as average values with standard deviation or as median with an interquartile range (25–75th percentile). Distribution was verified by the Kolmogorov–Smirnov test. Attribute variables were presented in the form of frequencies of individual categories. Statistical significance was estimated by the Chi-square test or Fisher’s exact test for attributive variables. Continuous variables were tested using Student’s *t*-test for (in) dependent samples or nonparametric alternative Mann–Whitney test and Wilcoxon test. The correlation was estimated by Pearson correlation. Predictive value exploration for the chosen variables in the assessment of adherence to MTX in the group of patients with RA was conveyed by univariate and multivariate logistic regression analyses. A *p*-value less than 0.05 for variables in the univariate analysis was used for inclusion in the multivariate analysis. The results of the logistic regression analysis were presented as odds ratio with 95% confidence interval (OR (95% CI). A *p*-value less than 0.05 was considered statistically significant for all analyses.

## 3. Results

Our study included 111 RA patients with an average age of 56.2 ± 10.6 years. Females made up the majority of the survey sample (78.4%), almost half of the patients (48.6%) were non-smokers, and 35.1% were employed. The majority of respondents lived in the city (79.3%) and had a high school education (68.5%) (Table 1). 

Comorbidities were marked in 74 (66.7%) patients, and 23 (20.7%) of them had three or more conditions. The median disease duration in our sample population from the establishment of diagnosis was 6 years (IQR: 3–13.5). The number of annual visits to a rheumatologist in our patients was 4 (IQR: 3–6), while the average laboratory analysis was conducted 4 times a year. 

In combined therapy adjacent to MTX, 96 patients (86.5%) were taking steroids and he median dose was 6 mg. Antimalarials were used by 33 (29.7%) patients, and 34 study participants (30.6%) were on biological therapy. A third of the subjects had side effects: gastrointestinal tract discomforts (13.5%), leukopenia, and elevated liver enzymes (10.8%).

According to the CDAI, low disease activity was detected in 29.7% of patients, moderate was observed in 32.4%, and high in 33.3%. Similar results were obtained by DAS28 scoring: 36.9% of study participants had high and 34.2% moderate disease activity. The average HAQ scale score was 0.89 ± 0.48.

The mental health analysis revealed that 18% of patients had elevated scores on the HADS scale for depression, while 10.8% of study participants had increased scores on the HADS scale for anxiety. About a quarter of patients accomplished a borderline HADS score for depression and anxiety.

Adherence to MTX measured by three scales (CQR19, MARS-5, and VAS) was represented as a dichotomous variable (Table 2). The average score for adherence to MTX on the CQR19 scale was 76.9 ± 13.5, while a non-adherence level to MTX was detected in 56 (50.5%) patients. According to the MARS-5 criteria, a non-adherence level was found in 32 study partakers (28.8%) (average score 22.63 ± 2.58). On the VAS ranging from 0 to 100 mm, patients graded self-reported adherence to MTX as 87.44 ± 16.49. Non-adherence to MTX (<80%) according to the VAS was detected in 19 of the study participants (17.1%) (Table 2).

**Figure 1:**
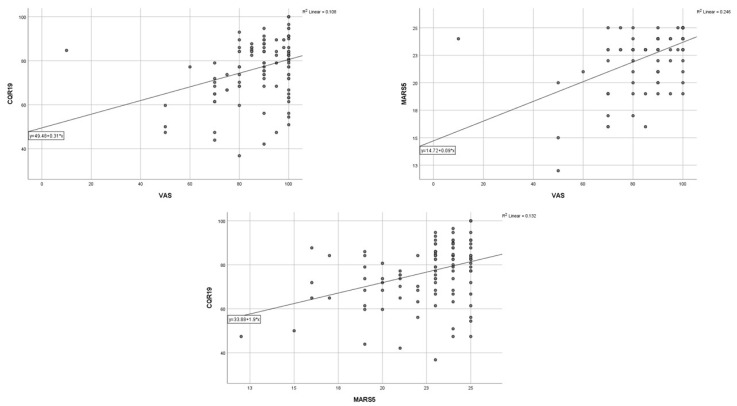
Correlation between three questionnaires of quality of life in patients with rheumatoid arthritis.

The analysis of all three scores used to measure adherence showed a statistically positive correlation of the CQR19 score with the MARS-5 score (r = 0.364, *p* < 0.001) and the VAS score (r = 0.329, *p* < 0.001). An even stronger positive correlation was observed between the VAS and MARS-5 scores (r = 0.496, *p* < 0.001) (Figure 1). 

In the analysis of adherence indicators, the CQR19, the MARS-5, and the VAS score identified significant differences in adherence to MTX regarding sex, age, and urban residence. Also, a prominent dissimilarity was observed with respect to the number of present chronic diseases, number of annual physician visits, MTX dose, and co-medication with steroids. Patients on corticosteroid therapy had markedly enhanced adherence expressed with the VAS (*p* = 0.049) and CQR19 score (*p* = 0.022) (Table 1). 

In the univariate logistic regression, statistical significance for non-adherence measured by the VAS was obtained for MTX dose (0.881 (0.783–0.992), *p* = 0.036) and concomitant steroid use (0.273 (0.078–0.956), *p* = 0.042). Patients who used a lower dose of MTX and those without concomitant steroid therapy had a higher degree of non-adherence (Table 3).

Using the MARS-5, statistical significance for non-adherence was obtained for place of residence (0.347 (0.134–0.901), *p* = 0.030), where a higher degree of non-adherence was obtained for those living in rural areas. 

Statistical significance was also obtained for the number of comorbidities (3.062 (1.057–8.874), *p* = 0.039), where patients with a higher number of comorbidities were more non-adherent. Additionally, the presence of depression (1.142 (1.010–1.293), *p* = 0.035) leads to a higher degree of non-adherence. As observed in the previous questionnaire, a statistically significant parameter for non-adherence was the concomitant use of steroids (0.306 (0.094–0.998). *p* = 0.050). This means that patients who were not using steroids had a higher degree of non-adherence (Table 3).

In the multivariate logistic regression, it was found that a statistically significant independent predictor for non-adherence was the concomitant use of steroids (Table 4). The concomitant use of corticosteroids reduces the chance of poor non-adherence by 4.4 times.

For non-adherence measured by the CQR scale by univariate regression, statistical significance was obtained for sex (0.264 (0.095–0.730), *p* = 0.010), where males have a higher degree of non-adherence. Statistical significance was detected both for age (1.041 (1.003–1.081), *p* = 0.034), meaning older patients were more non-adherent, as well as for the concomitant use of steroids (0.157 (0.033–0.746), *p* = 0.020).

The multivariate logistic regression identified gender and the concomitant use of steroids as statistically significant independent predictors of non-adherence (Table 5). The concomitant use of corticosteroids diminishes the odds of non-adherence by 5.1 times, while the female gender reduces this chance by approximately 3.9.

Finally, the composite score is shown as non-adherence in at least two of the three tested scores. The multivariate logistic regression identified comorbidities and the concomitant use of steroids as statistically significant predictors of non-adherence (Table 6). The concomitant use of corticosteroids diminishes the odds of non-adherence by 4.65 times, while a smaller number of comorbidities reduces this chance by approximately 4.1.

## 4. Discussion

In this study, the employment of three self-assessment questionnaires showed that 48.6% (CQR19), 70.3% (MARS-5), and 82.9% (VAS) of patients were adherent. The logistic regression showed that the concomitant use of corticosteroids is an independent predictor of adherence to MTX in all three questionnaires. Current studies exhibit significant variation in the degree of adherence using different measuring instruments. The latest research on adherence assessment by the CQR questionnaire found that 78% and 85.7% of patients were adherent [13,35]. De Cuyper et al. demonstrated that only 24.2% of patients were adherent when the MARS-5 questionnaire estimation tool was used, while the VAS score delivered more promising results, showing adherent behavior in 94% of the subjects. This observation can be explained by the absence of an existing standard for adherence measurement and the probability of overestimation in the self-reporting approach. The CQR was chosen as the only validated questionnaire in the rheumatology field. For the MARS-5 questionnaire, the latest studies did not use the same cut-off point for the dichotomization of the MARS-5, limiting the possibility of data comparison.

The statistical difference was detected between adherent and non-adherent patients in terms of gender, age, place of residence, number of annual visits to a physician, MTX dose, and steroid co-medication. In our cohort, younger patients had a higher degree of adherence, which is consistent with the data from van den Bemt et al. [15].

This could be clarified by the association of older age with comorbidities and therefore polypharmacy, increasing the probability of side effect incidence. However, other research groups report opposing results that show adherence in the elderly patient population [36,37,38], succeeding to the conclusion that age can affect adherence differentially. The female gender impact on better adherence was previously observed [39,40,41], while some authors report inconsistency [16,35].

The positive influence of comorbidities on non-adherence is in accordance with published research [35,40], where two or more comorbidities represent a predictor of non-adherence. When multiple comorbidities are present, complexity and the extent of therapy carry a risk of forgetfulness and unintentional non-adherence [42]. In our study, patients who had a higher number of annual visits to a physician were more adherent. This is understandable since doctor–patient relations have a big effect on the efficacy of therapeutic regimens. Occasionally, non-adherence can occur due to a lack of conscientiousness in patients regarding medication necessity [43]. Educating patients about their condition and therapy, as well as the doctor–patient confidence level, can diversly affect adherence [44]. 

In the univariate logistic regression following the VAS, the current MTX dose was a significant predictor of non-adherence, while the MARS-5 questionnaire showed a marked prediction of adherence for depression and comorbidities. This was also shown for gender and age in the CQR19.

The concomitant use of steroids was a statistically significant predictor of non-adherence, according to the results of all three questionnaires. The majority of studies did not find an association between the dose of MTX and adherence, and only one study highlighted a connection between the higher dose of MTX with improved adherence [45], but the supportive validation from other research groups is missing [35,46].

The negative association between worse mental health and adequate adherence has also been shown in studies by other authors [13,47], which is consistent with our research. Patients suffering from depression have a 2–3 times higher rate of non-adherence compared to those without this disorder [44]. 

In our study, the multivariate analysis revealed that the concomitant use of steroids is an independent positive predictor of adherence, measured by the MARS-5 and CQR19. According to our knowledge, the influence of the concomitant use of steroids on MTX adherence was analyzed in only a few studies and the results are inconsistent [48,49,50]. For example, H. Bliddal et al. did not find any influence of prednisolone therapy on MTX adherence [49], while the negative impact of concomitant steroid use was shown by Alrubaye et al. [50]. On the other hand, Hoekstra M et al. showed that the concomitant use of prednisolone remained significantly related to MTX therapy in a multivariate analysis, which is consistent with our results [48]. 

This result may be a consequence of the fact that our patients had more frequent relapses of the disease, which required the use of steroids and thus affected the patients’ belief in the necessity of MTX therapy. Perhaps the large number of patients who received steroids in comparison with other studies where this number was significantly smaller influenced this result. 

Several research studies showed that the lack of comprehension related to MTX’s slow-acting effects can impact adherence [50]. Presumably, steroid administration with rapid and efficient action could have altered adherence. Treharna et al. found that adherence was better in a group of patients taking steroids because the absence of therapy can lead to a significant worsening of symptoms in the active form of the disease [51]. These results could also be the explanation for the better adherence to MTX in our group of patients on steroids, where steroids enhance the importance of MTX itself in the treatment of RA. On the other hand, the side effects of steroids cause concern in patients and could be diminished with MTX as a steroid-sparing drug.

Since our results show that the concomitant use of steroids can increase adherence to MTX, this may have a clinical implication in patients who have discontinued steroids (due to less disease activity), as they require additional attention in their adherence to MTX therapy.

Since all three scores showed a good correlation, a composite score was created. It showed similar results as for each score individually. Therefore, it can be recommended that each of these three scores can be equally used separately or together in the same patient. This is very important for the further clinical implication of these adherence tests.

The main limitations of this study are its cross-sectional design and monocentric character. A cross-sectional design enables adherence-related variable identification but with inconclusive results. An additional problem is a bias selection since all included subjects were recruited from the same institution and had an opportunity for more frequent follow-ups, which could have diminished the inclusion of non-adherent patients. Also, a small patient sample size does not have enough statistical power to determine the influence of the examined variables on adherence. The utilization of the MARS-5 questionnaire without a clearly defined cut-off point for the separation of adherent and non-adherent study participants may result differentially. Commonly, the use of scale dichotomization and cut-off points can lead to the loss of important information due to the simplification of the examined characteristics. Also, employing self-assessment scales can result in the overestimation of adherence due to social desirability and giving adjusted answers. The research did not convey a follow-up over time, and it is known that adherence is a dynamic process that can be influenced by several factors and that patient behavior can change over time.

This study’s strength is using three measuring scales that exhibited significant mutual correlation, especially that observed between the VAS and MARS-5. To our knowledge, DeCuyper et al. were the only other authors that used all three self-reported questionnaires assessing adherence to MTX, and they have shown similar results. In accordance with the research of these authors, this would indicate the importance of VAS application in everyday use for the rapid assessment of adherence to MTX in patients with RA [13].

## 5. Conclusions

Our study showed that a significant percentage of patients with RA are non-adherent. Furthermore, extensive variation in the level of adherence can be observed depending on the measuring tool. A curious fact is that the concomitant use of steroids was singled out as an independent predictor of non-adherence. Since adherence to MTX in patients with RA is still suboptimal and measurements are not standardized, they are nevertheless necessary for daily clinical practice. The use of self-reporting questionnaires may have clinical relevance for the rapid detection of adherence. Given the above facts, further research is needed to discover non-adherent patients and to explain the reasons for non-adherence, as well as to develop adapted and possibly highly personalized interventions to improve adherence. 

## Figures and Tables

**Table 1:** Participant characteristics and comparison between demographic, clinical, and psychological features of adherent and non-adherent patients.

	All (n = 111)	VAS			MARS-5			CQR19		
Variable		Adherent	Non-Adherent	*p*	Adherent	Non-Adherent	*p*	Adherent	Non-Adherent	*p*
Female n (%)	87 (78.4)	72 (78.3)	15 (78.9)	1	64 (82.1)	22 (68.8)	0.201	48 (88.9)	38 (67.9)	0.015
Male n (%)	24 (21.6)	20 (21.7)	4 (21.1)		14 (17.9)	10 (31.3)		6 (11.1)	18 (32.1)	
Age (yrs)	56.2 ± 10.6	56.0 ± 9.9	57.1 ± 13.8	0.708	56.3 ± 10.5	56.5 ± 10.9	0.927	54.2 ± 10.1	58.5 ± 10.6	0.031
Living city, n (%)	88 (79.3)	73 (79.3)	15 (78.9)	1	66 (84.6)	21 (65.6)	0.049	43 (79.6)	44 (78.6)	1
Living countryside, n (%)	23 (20.7)	19 (20.7)	4 (21.1)		12 (15.4)	11 (34.4)		11 (20.4)	12 (21.4)	
Employment status										
Unemployed n (%)	31 (27.9)	27 (29.3)	4 (21.1)	0.564	23 (29.5)	8 (25.0)	0.861	18 (33.3)	13 (23.3)	0.24
Employed n (%)	39 (35.1)	33 (35.9)	6 (31.6)		27 (34.6)	11 (34.4)		20 (37.0)	18 (32.1)	
Retiree n (%)	41 (36.9)	32 (34.8)	9 (47.7)		28 (35.9)	13 (40.6)		16 (29.6)	25 (44.6)	
Education										
Primary n (%)	9 (8.1)	7 (7.6)	2 (10.5)	0.896	5 (6.4)	4 (12.5)	0.528	3 (5.6)	6 (10.7)	0.539
Secondary n (%)	76 (68.5)	63 (68.5)	13 (68.4)		55 (70.5)	20 (62.5)		39 (72.2)	36 (64.3)	
Higher n (%)	26 (23.4)	22 (23.9)	4 (21.1)		18 (23.1)	8 (25.0)		12 (22.2)	14 (25.0)	
Non-smoker n (%)	54 (48.6)	44 (47.8)	10 (52.6)	0.742	38 (48.7)	15 (46.9)	0.936	22 (40.7)	31 (55.4)	0.308
Ex-smoker n (%)	25 (22.5)	22 (23.9)	3 (15.8)		17 (21.8)	8 (25.0)		14 (25.9)	11 (19.6)	
Smoker n (%)	32 (28.8)	26 (28.3)	6 (31.6)		23 (29.5)	9 (28.1)		18 (33.3)	14 (25.0)	
Comorbidities: n (%)										
0	37 (33.3)	32 (34.8)	5 (26.3)	0.373	28 (35.9)	8 (25.0)	0.039	22 (40.7)	14 (25.0)	0.243
1	30 (27.0)	22 (23.9)	8 (42.1)		16 (20.5)	14 (43.8)		12 (22.2)	18 (32.1)	
2	21 (18.9)	19 (20.7)	2 (10.5)		14 (17.9)	7 (21.9)		8 (14.8)	13 (23.2)	
≥3	23 (20.7)	19 (20.7)	4 (21.1)		20 (26.6)	3 (9.4)		12 (22.2)	11 (19.6)	
Disease duration (yrs)	6 (3–13.5)	6 (3–13)	7 (4–17)	0.452	6 (3–13.8)	6 (3.3–13.5)	1	6 (3.8–14.3)	6 (3–10.3)	0.37
Physician visits (per year)	4 (3–6)	4 (3–6)	3 (2–4)	0.036	4 (3–6)	3 (3–7.5)	0.285	4 (3–6)	4 (3–5.8)	0.812
Tender joint count (n)	5 (1–10)	5 (1–10)	4 (0–12)	0.488	6 (1–10)	4 (0–9.5)	0.298	4.5 (1–10)	5 (1–11)	0.797
Swollen joint count (n)	0 (0–3)	0 (0–2.5)	0 (0–4)	0.635	1 (0–2)	0 (0–3.8)	0.629	0.5 (0–2.8)	0 (0–3)	0.869
ESR (mm/h)	26 (14–52)	26 (14–51)	39 (20–63)	0.187	26 (12–51)	28 (17.5–72.75)	0.293	24 (13–47.5)	28 (14.75–62.25)	0.154
CRP (mg/L)	3.89 (1.28–14.99)	3.42 (1.00–13.96)	4.75 (2.98–18.3)	0.197	3.05 (0.92–10.03)	6.5 (2.39–15.73)	0.062	2.87 (0.87–9.53)	5.21 (2–19)	0.042
IL-6 (pg/mL)	12.7 (3.59–42.26)	14.34 (3.23–13.96)	5.38 (4.43–42.2)	0.599	12.08 (2.89–42.26)	14.2 (4.03–41.75)	0.832	16.68 (7.76–43.24)	4.7 (2–40.34)	0.059
Dosage MTX (mg)	15 (10–17.5)	15 (10–17.5)	12.5 (10–15)	0.03	15 (10–17.5)	12.5 (10–15)	0.22	15 (10–17.5)	15 (10–17.5)	0.506
Side effects of MTX, n (%)	31 (28.2)	23 (25.0)	8 (42.1)	0.218	20 (25.6)	11 (34.4)	0.489	12 (22.2)	19 (33.9)	0.249
Concomitant steroids, n (%)	96 (86.5)	82 (91.1)	14 (73.7)	0.049	70 (92.1)	25 (78.1)	0.086	51 (96.2)	44 (80.0)	0.022
Dosage of corticosteroids (mg)	6.0 (5–9.5)	5.5 (5–8.5)	7.5 (5–10)	0.64	5 (5–8.5)	7.5 (5–10)	0.473	6 (5–10)	5.5 (5–9.5)	0.659
sDMARD n (%)	44 (39.6)	35 (38.0)	9 (47.4)	0.618	26 (33.3)	17 (53.1)	0.086	17 (31.5)	26 (46.4)	0.158
bDMARD n (%)	34 (30.6)	30 (32.6)	4 (21.1)	0.471	24 (30.8)	10 (31.2)	1.000	16 (29.6)	18 (32.1)	0.937
CDAI	17.7 ± 12.4	17.8 ± 12.8	17.3 ± 10.3	0.894	17.9 ± 12.7	16.97 ± 11.8	0.732	16.4 ± 11.5	18.7 ± 13.2	0.347
DAS28-ESR	4.31 ± 1.7	4.28 ± 1.8	4.4 ± 1.5	0.761	4.3 ± 1.7	4.3 ± 1.8	0.871	4.2 ± 1.7	4.4 ± 1.7	0.628
HAQ	0.9 ± 0.5	0.9 ± 0.5	0.9 ± 0.4	0.881	0.9 ± 0.5	0.9 ± 0.4	0.935	0.9 ± 0.5	0.9 ± 0.5	0.694
HADS depression	7.6 ± 3.5	7.4 ± 3.5	9.0 ± 3.7	0.072	7.1 ± 3.5	8.8 ± 3.3	0.03	7.2 ± 3.4	7.9 ± 3.7	0.246
HADS anxiety	6.1 ± 3.8	5.8 ± 3.7	7.6 ± 4.0	0.07	6.0 ± 4.0	6.2 ± 3.1	0.776	6.6 ± 4.1	5.6 ± 3.4	0.204

Results are expressed as mean value ± standard deviation, median (25th; 75th percentile), or number (percentage). Chi-square test or Fisher’s exact test; Mann–Whitney test; Independent Samples *t*-test. VAS—Visual Analogue Scale; CQR19—Compliance-Questionnaire-Rheumatology; MARS-5—Medication Adherence Reports Scale; ESR—erythrocyte sedimentation rate; CRP—C reactive protein; IL-6—interleukin 6; MTX—methotrexate; sDMARDs—synthetic disease-modifying antirheumatic drugs; bDMARDs—biological disease-modifying antirheumatic drugs; CDAI—Clinical Disease Activity Index; DAS28-ESR—Disease Activity Score 28-joint count; HAQ—Health Assessment Questionnaire; HADS—Hospital Anxiety and Depression Scale.

**Table 2:** The medication adherence rate to methotrexate.

Scale		Adherence	N (%)
CQR19	76.92 ± 13.47	Non-adherent	56 (50.5)
		Adherent	54 (48.6)
MARS-5	22.63 ± 2.58	Non-adherent	32 (28.8)
		Adherent	78 (70.3)
VAS	87.44 ± 16.49	Non-adherent	19 (17.1)
		Adherent	92 (82.9)

Results are expressed as mean value ± standard deviation or number (percentage) (N (%); adherence is expressed as a dichotomous variable. VAS—Visual Analogue Scale; CQR19—Compliance-Questionnaire-Rheumatology; MARS-5—Medication Adherence Reports Scale.

**Table 3:** Univariate logistic regression: prediction of non-adherent behavior.

Baseline Predictor	VAS		MARS-5		CQR19	
	OR (95%CI)	*p*	OR (95%CI)	*p*	OR (95%CI)	*p*
Gender	1.042 (0.311–3.490)	0.947	0.481 (0.187–1.238)	0.481	0.264 (0.095–0.730)	0.010 *
Age (yrs)	1.009 (0.963–1.057)	0.705	1.002 (0.963–1.042)	0.926	1.041 (1.003–1.081)	0.034 *
Residence	0.976 (0.209–3.283)	0.969	0.347 (0.134–0.901)	0.030 *	0.938 (0.374–2.353)	0.891
Employment status	1.227 (0.314–4.799)	0.768	1.171 (0.403–3.405)	0.772	1.246 (0.479–3.242)	0.652
Education	0.722 (0.134–3.879)	0.704	0.455 (0.111–1.863)	0.273	0.462 (0.107–1.984)	0.299
Tobacco use	0.600 (0.150–2.404)	0.471	1.192 (0.425–3.343)	0.738	0.558 (0.213–1.457)	0.233
Comorbidities	2.327 (0.672–8.060)	0.183	3.062 (1.057–8.874)	0.039 *	2.357 (0.875–6.351)	0.090
Disease duration (years)	1.027 (0.967–1.092)	0.385	0.997 (0.944–1.053)	0.907	0.973 (0.925-1.023)	0.286
Physician visits (per year)	0.904 (0.759-1.077)	0.260	1.006 (0.889–1.137)	0.929	1.007 (0.899-1.127)	0.910
Tender joint count	0.974 (0.892-1.062)	0.549	0.974 (0.907–1.046)	0.464	0.996 (0.936–1.061)	0.913
Swollen joint count	1.001 (0.841–1.191)	0.992	1.007 (0.871–1.163)	0.930	1.039 (0.908–1.189)	0.579
ESR (mm/h)	1.009 (0.995–1.023)	0.226	1.005 (0.992–1.017)	0.473	1.008 (0.996–1.020)	0.186
CRP (mg/L)	1.001 (0.978–1.024)	0.947	0.992 (0.971–1.014)	0.498	1.005 (0.987–1.023)	0.587
IL-6 (pg/mL)	0.982 (0.948–1.018)	0.327	1.003 (0.986–1.021)	0.727	0.997 (0.981–1.013)	0.711
Dosage MTX (mg)	0.881 (0.783–0.992)	0.036 *	0.952 (0.864–1.048)	0.314	0.981 (0.889–1.071)	0.673
Concomitant steroids	0.273 (0.078–0.956)	0.042 *	0.306 (0.094–0.998)	0.050 *	0.157 (0.033–0.746)	0.020 *
Dosage of steroids (mg)	1.019 (0.829–1.252)	0.857	1.067 (0.905–1.258)	0.440	0.995 (0.859–1.154)	0.951
sDMARDs	1.303 (0.470–3.615)	0.611	2.267 (0.980–5.244)	0.056	1.886 (0.866–4.107)	0.110
bDMARDs	0.677 (0.205–2.231)	0.521	1.062 (0.424–2.662)	0.898	1.143 (0.493–2.649)	0.756
Side effects of MTX	2.182 (0.782–6.085)	0.136	1.519 (0.624–3.696)	0.357	1.797 (0.770–4.193)	0.175
CDAI	0.997 (0.957–1.039)	0.893	0.994 (0.960–1.029)	0.729	1.016 (0.983–1.049)	0.344
DAS 28-ESR	1.047 (0.781–1.404)	0.759	0.980 (0.768–1.250)	0.869	1.058 (0.846–1.323)	0.624
VAS	0.999 (0.976–1.022)	0.942	1.006 (0.986–1.025)	0.574	1.011 (0.993–1.029)	0.246
HAQ	0.920 (0.315–2.690)	0.879	1.038 (0.434–2.482)	0.934	1.179 (0.524–2.654)	0.691
HADS depression	1.137 (0.986–1.311)	0.077	1.142 (1.010–1.293)	0.035 *	1.068 (0.956–1.193)	0.245
HADS anxiety	1.126 (0.988–1.283)	0.076	1.017 (0.909–1.137)	0.773	0.934 (0.842–1.038)	0.204

* Significant result that is included in the multivariable analysis; VAS—Visual Analogue Scale; CQR19—Compliance-Questionnaire-Rheumatology; MARS-5—Medication Adherence Reports Scale; OR (95%CI)—odds ratio (95% confidence interval); ESR—erythrocyte sedimentation rate; CRP—C reactive protein; IL-6—interleukin 6; MTX—methotrexate; sDMARDs—synthetic disease-modifying antirheumatic drugs; bDMARDs—biological disease-modifying antirheumatic drugs; CDAI—Clinical Disease Activity Index; DAS28-ESR—Disease Activity Score 28-joint count; HAQ—Health Assessment Questionnaire; HADS—Hospital Anxiety and Depression Scale.

**Table 4:** Multivariate logistic regression analysis: predictors of non-adherence measured by the MARS-5.

Predictor	OR (95%CI)	*p*
HADS depression	1.131 (0.994–1.286)	0.061
Concomitant use of steroids	0.281 (0.078–0.999)	0.050
Comorbidities	0.805 (0.538–1.206)	0.293

OR (95%CI)—odds ratio (95% confidence interval); HADS—Hospital Anxiety and Depression Scale; MARS-5—Medication Adherence Reports Scale.

**Table 5:** Multivariate logistic regression analysis: predictors of non-adherence measured by the CQR19.

Predictor	OR (95%CI)	*p*
Gender	0.256 (0.088–0.742)	0.012
Age	1.038 (0.996–1.082)	0.075
Concomitant use of steroids	0.196 (0.039–0.992)	0.049

OR (95%CI)—odds ratio (95% confidence interval); CQR19—Compliance-Questionnaire-Rheumatology.

**Table 6:** Univariate and multivariate logistic regression analysis: predictors of non-adherence measured by the composite score #.

Baseline Predictor	Univariate Logistic Regression		Multivariate Logistic Regression	
	OR (95%CI)	*p*	OR (95%CI)	*p*
Gender	0.382 (0.149–0.979)	0.045 *	0.422 (0.150–1.189)	0.103
Age (yrs)	1.014 (0.975–1.055)	0.477		
Residence	0.559 (0.213–1.465)	0.237		
Employment status	1.027 (0.348–3.027)	0.962		
Education	0.271 (0.066–1.116)	0.071		
Tobacco use	1.311 (0.464–3.704)	0.609		
Comorbidities	4.143 (1.389–12.352)	0.011 *	4.139 (1.321–13.063)	0.015
Disease duration (years)	0.982 (0.929–1.037)	0.514		
Physician visits (per year)	0.982 (0.866–1.113)	0.772		
Tender joint count	0.984 (0.917–1.055)	0.650		
Swollen joint count	1.034 (0.897–1.191)	0.647		
ESR (mm/h)	1.011 (0.998–1.023)	0.092		
CRP (mg/L)	1.001 (0.982–1.020)	0.941		
IL-6 (pg/mL)	1.001 (0.983–1.018)	0.940		
Dosage MTX (mg)	0.972 (0.883–1.069)	0.557		
Concomitant steroids	0.208 (0.062–0.698)	0.011 *	0.215 (0.059–0.782)	0.020
Dosage of steroids (mg)	1.082 (0.916–1.278)	0.354		
sDMARDs	1.821 (0.792–4.187)	0.158		
bDMARDs	1.042 (0.429–2.532)	0.928		
Side effects of MTX	1.545 (0.636–3.757)	0.337		
CDAI	1.001 (0.968–1.035)	0.958		
DAS 28-ESR	1.046 (0.819–1.335)	0.720		
VAS	1.007 (0.988–1.027)	0.488		
HAQ	1.141 (0.477–2.733)	0.766		
HADS depression	1.118 (0.990–1.262)	0.071		
HADS anxiety	1.007 (0.901–1.125)	0.908		

* Significant result that is included in the multivariable analysis; #—composite score implies that patients are marked as non-adherent if non-adherence is shown in at least two of the three tested scores; OR (95%CI)—odds ratio (95% confidence interval); ESR—erythrocyte sedimentation rate; CRP—C reactive protein; IL-6—interleukin 6; MTX—methotrexate; sDMARDs—synthetic disease-modifying antirheumatic drugs; bDMARDs—biological disease-modifying antirheumatic drugs; CDAI—Clinical Disease Activity Index; DAS28-ESR—Disease Activity Score 28-joint count; HAQ—Health Assessment Questionnaire; VAS—Visual Analogue Scale; HADS—Hospital Anxiety and Depression Scale.

## Data Availability

The data sets used and/or analyzed in the present study are available from the corresponding author on reasonable request.
